# To Readers, Correspondents and Authors

**Published:** 1828-04

**Authors:** 


### READERS, CORRESPONDENTS AND AUTHORS.

I. It is not without misgivings that the Editor inserts the discourse on Intemperance, which makes the first article of this number. Its declamatory style, and the familiar acquaintance of the profession with most of the facts and principles which it embraces, would seem to render its introduction into a medical journal almost inexcusable—but still, the editorial offence is perhaps venial. As it respects the preservation of health, few subjects are of greater moment than temperance; every physician, therefore, should labour to promote it. To this end he ought thoroughly to study all the causes and consequences of Intemperance. These are moral as well as physical; and if he does not extend his inquiries to both, his influence on society must of necessity be exceedingly limited. Any attempt, however, to depict the moral consequences of this vice, as a dissuasive against the practice of it, naturally carries both speaker and auditor into regions of declamation and rhetorick. To write such a discourse, expressly for insertion in a journal of the medical sciences, would be a different thing, from the determination to place it there, after being publickly pronounced, on the occasion for which it was prepared It was not easy to divest it of the attributes of a popular address, or reduce it to a condition of scientific gravity; and hence the Editor found it necessary, either to publish it *in extenso*, or to exclude it altogether. Many of his senior readers may wish he had adopted the latter alternative; but his junior brethren, for whom the journal is more especially designed, will perhaps, in general, be pleased that he has chosen the former.

II. Desirous of publishing quarterly reports, of the weather and diseases of the different parts of the Western Country, the Editor respectfully solicits from his friends, and especially his former pupils, communications of that kind. He would wish them to be brief; and, if the expression may be allowed, graphical; divested, as far as possible, of common place facts; and possessed of a decided specific character. They should relate to the respective seasons—Summer, Winter, Spring and Autumn. When published in one journal, the series will constitute a synopsis of the history of the epidemic diseases of the West and South. They will be inserted under the head of Original Intelligence.

III. It will be seen that a department of Bibliographical notices has been established, in which the Editor will give a short account of the various new professional works that may fall into his hands;—a design to which he respectfully invites the attention of Booksellers, Publishers and Authors.

